# Fast‐track endovascular aortic repair: Interim report from the prospective LIFE registry

**DOI:** 10.1002/ccd.26626

**Published:** 2016-07-12

**Authors:** Zvonimir Krajcer, Venkatesh G. Ramaiah, Meredith Huetter, Larry E. Miller

**Affiliations:** ^1^ St. Luke's Episcopal HospitalHoustonTexas; ^2^ Arizona Heart InstitutePhoenixArizona; ^3^ TriVascular, IncSanta RosaCalifornia; ^4^ Miller Scientific Consulting, IncAshevilleNorth Carolina

**Keywords:** aortic disease, aortic repair, endovascular, endovascular intervention

## Abstract

**Objective:**

To assess the feasibility, safety, and clinical utility of a fast‐track endovascular aneurysm repair (EVAR) protocol.

**Background:**

Despite recent advances in EVAR technology and techniques, considerable opportunity exists to further improve EVAR efficiency and outcomes.

**Methods:**

Eligible patients underwent elective EVAR with the Ovation Prime stent graft. Successful completion of the fast‐track protocol required bilateral percutaneous access, avoidance of general anesthesia and intensive care unit admission, and next‐day discharge. Patients were followed through 1‐month post‐treatment.

**Results:**

Between October 2014 and September 2015, 129 patients were enrolled in the study. Vascular access, stent graft delivery, and stent graft deployment were successful in all patients. The fast‐track EVAR protocol was successfully completed in 114 (88%) patients. Bilateral percutaneous access was achieved in 97% of cases. Comparing patients who completed fast‐track requirements to those who failed at least one component, procedure time was 86 vs. 122 min, use of general anesthesia was 0% vs. 20%, need for intensive care unit stay was 0% vs. 13%, hospital stay was 1.1 vs. 2.1 days, and postoperative groin pain severity (0–10 scale) was 1.2 vs. 4.0. No type I or III endoleaks, serious device‐related adverse events, AAA ruptures, surgical conversions, or AAA‐related secondary procedures were reported. One (0.9%) patient in the fast‐track group died from acute respiratory failure.

**Conclusions:**

Initial results from the LIFE study are encouraging and suggest that a fast‐track protocol is feasible, safe, and may improve efficiency of healthcare resource allocation in select patients undergoing EVAR. © 2016 Wiley Periodicals, Inc.

## INTRODUCTION

Endovascular aortic repair (EVAR) is associated with lower perioperative morbidity and mortality rates compared to open surgical resection 1 and has become the standard of care for treatment of abdominal aortic aneurysm (AAA) requiring intervention. As EVAR technology continues to advance in an effort to address unmet clinical needs such as improving treatment durability, reducing need for secondary interventions, and expanding patient eligibility, there is also considerable opportunity to reduce perioperative healthcare utilization with EVAR. Traditional EVAR involves vascular access via femoral cutdown, general anesthesia, overnight intensive care unit (ICU) stay, and a 3‐day hospital stay 2. In the current economic climate with heightened scrutiny on health care resource utilization, adoption of hospital care pathways aimed at accelerating patient recovery and reducing perioperative morbidity should be considered. For open aortic surgery, such fast‐track initiatives have been adopted with increasing frequency 3, 4, resulting in shorter ICU and hospital stays and reductions in morbidity compared to traditional surgery 5, 6, 7, 8, 9. Although the potential clinical and cost benefits associated with fast‐track EVAR are substantial, experience with fast‐track EVAR pathways remains limited. A randomized controlled trial of totally percutaneous EVAR using suture‐mediated closure devices demonstrated shorter time to hemostasis, faster procedure times, and noninferior perioperative outcomes versus standard open femoral exposure 10. The largest study utilizing a fast‐track EVAR protocol involved 915 patients treated with bilateral percutaneous access and local anesthesia/conscious sedation 11. Treatment success was achieved in 94% of cases, mean hospital stay was 1.3 days, and 30‐day mortality was only 0.6%. It is plausible that development of a least‐invasive fast‐track EVAR protocol could further improve upon these promising outcomes. The Least Invasive Fast‐Track EVAR (LIFE) registry was developed to explore the clinical utility and resource utilization of a defined fast‐track EVAR protocol in patients undergoing elective AAA repair with an ultra low‐profile stent graft. We report herein perioperative outcomes from the first 129 patients enrolled in the prospective multicenter LIFE registry.

## METHODS

The LIFE registry is a prospective, nonrandomized, multicenter post‐market study designed to evaluate the feasibility, safety, and clinical utility of a least‐invasive fast‐track EVAR protocol in 250 patients with AAA requiring intervention. The study was approved by the institutional review board at each participating site and all patients provided informed written consent before study participation. The study was prospectively registered at www.clinicaltrials.gov (NCT02224794).

### Participants

Eligible patients were adults with AAA requiring elective intervention with anatomy suitable for endovascular repair. The main inclusion and exclusion criteria are reported in Table 1. Pretreatment assessments included medical and surgical history, laboratory tests, and spiral contrast‐enhanced computed tomography. Patient enrollment was conditional on the investigator determining that bilateral percutaneous access, avoidance of general anesthesia and intensive care unit stay, and next‐day hospital discharge were feasible and did not jeopardize patient safety. Following enrollment, patients remained in the study through the 1‐month follow‐up visit, regardless of whether all components of the fast‐track protocol were completed.

**Table 1:** Main Study Entry Criteria

**Main inclusion criteria**
• Age ≥ 18 years
• Male or nonpregnant female
• Candidate for elective open surgical AAA repair
• AAA >5.0 cm diameter, increased ≥0.5 cm diameter in last 6 months, or maximum diameter >1.5× adjacent non‐aneurysmal aorta
• Suitable anatomy for endovascular repair with the Ovation Prime stent graft
• Suitable anatomy to allow Perclose Proglide suture‐mediated closure system via the pre‐close technique

**Main exclusion criteria**
• Dissecting or acutely ruptured AAA
• Acute vascular injury
• Prior AAA or iliac artery repair
• Mycotic AAA or active systemic infection
• Unstable angina
• Unstable peripheral artery disease with critical limb ischemia
• Congestive heart failure
• Myocardial infarction or stroke within the past 3 months
• Need for renal artery coverage (e.g., Chimney graft)
• Planned adjunctive devices (e.g., renal stent)
• Major surgery or interventional procedure within the past 30 days
• Connective tissue disease (e.g., Marfan's or Ehler's–Danlos syndrome)
• History of bleeding disorder or refuses blood transfusions
• Dialysis‐dependent renal failure or serum creatinine >2.0 mg dL^−1^
• Morbid obesity (BMI ≥40 kg m^−2^)
• Home oxygen use
• Patient admitted from skilled nursing facility
• Life expectancy <1 year
• Anticipated inability to discharge patient within 1 day
• Participation in investigational device or drug clinical trial
• Intolerance/hypersensitivity to anticoagulation, contrast media, or stent graft components

### Device

Patients underwent elective EVAR with the Ovation Prime Abdominal Stent Graft System (TriVascular, Santa Rosa, CA). The aortic body is delivered through a flexible hydrophilic‐coated 14 Fr OD catheter, which is ideal for bilateral percutaneous access. The aortic body is comprised of a low permeability PTFE graft and a suprarenal nitinol stent with integral anchors to achieve active fixation to the aortic wall. The aortic body contains a network of inflatable channels and sealing rings that are filled during deployment with a low viscosity, radiopaque fill polymer that cures *in situ* to create a conformable seal to the aortic neck. The Ovation Prime iliac limbs are comprised of highly flexible nitinol stents encapsulated in low‐permeability PTFE that are packaged in ultra‐low profile 13‐14F OD delivery system.

### Outcomes

Outcomes of the LIFE registry included ability to successfully complete all components of the fast‐track protocol, procedural details, convalescence, device‐related complications, major adverse events, postoperative groin pain, and health‐related quality of life. Major adverse events included death, myocardial infarction, stroke, renal failure, respiratory failure, paralysis, bowel ischemia, and procedural blood loss ≥1,000 cm^3^. Adverse events were adjudicated by a clinical events committee (CEC). Postoperative groin pain was measured with the Wong–Baker FACES Pain Rating Scale, ranging from 0 (none) to 10 (worst imaginable) 12. Health‐related quality of life was measured with the EQ‐5D 13, which provides a single index value for health status and is comprised of five dimensions including mobility, self‐care, usual activities, pain/discomfort, and anxiety/depression. The values for each EQ‐5D dimension are converted to a weighted health state index ranging from 0 (death) to 1 (perfect health) using population norms. Patients in the LIFE registry were followed through the 1‐month follow‐up visit since the primary benefits of a fast‐track EVAR program were anticipated to be realized in the perioperative period.

### Statistical Analysis

All patients enrolled in the LIFE registry were included in the statistical analysis, regardless of whether the fast‐track protocol was completed successfully. Enrollment in this study is ongoing and, therefore, hypothesis testing was not performed. Planned statistical methods and hypotheses based on final study data are described elsewhere 14. Descriptive comparisons of patients who did or did not complete the fast‐track EVAR protocol are provided. Continuous data were reported using mean and standard deviation or median, minimum, and maximum, depending on normality assumptions. Categorical data were reported with percentages. Changes in EQ‐5D over the 1‐month follow‐up in each group were assessed with paired samples t‐tests. All analyses were conducted in SAS v. 9.4 (SAS Institute, Cary, NC).

## RESULTS

**Figure 1:**
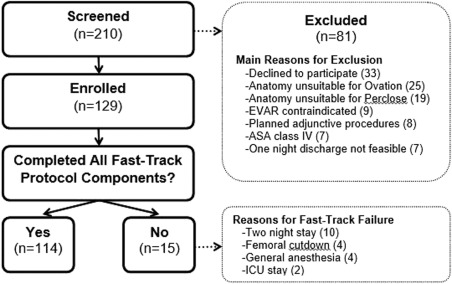
Patient flow diagram. ASA: American Society of Anesthesiologists; EVAR: endovascular aneurysm repair; ICU: intensive care unit. Sum of reasons for exclusion and fast‐track failure exceed number of patients in each respective category due to multiple reasons in some patients.

Between October 2014 and September 2015, 210 patients were screened and 129 patients were enrolled in the study. Main reasons for study ineligibility are detailed in Fig. 1. Enrollment in the LIFE registry is ongoing with planned enrollment of 250 patients anticipated by mid‐2016. Only 12% of screened patients were anatomically unsuitable for the Ovation stent graft and 9% were unsuitable for Perclose closure (mainly due to access vessel diameter <5 mm). Bilateral percutaneous access was attained in 97% of cases. The fast‐track EVAR protocol was successfully completed in 88% of enrolled patients.

Of the 129 study participants, 87% were men and mean age was 73 years. The most common risk factors were hyperlipidemia (81%), tobacco history (74%), hypertension (74%), and coronary artery disease (43%) (Table 2). Aortoiliac characteristics included mean AAA diameter of 51 mm, neck length of 25 mm, neck angulation of 22 degrees, and external iliac diameters of 7.8 mm (Table 3).

**Table 2:** Baseline Patient Characteristics

Variable	Fast track (*n* = 114)	Non‐fast track (*n* = 15)
Male gender	87%	87%
Age (years)^a^	73 ± 7	74 ± 10
ASA classification		
I/II	29%	13%
III	71%	87%
Hyperlipidemia	83%	67%
Tobacco history	75%	73%
Hypertension	72%	87%
Coronary artery disease	44%	33%
COPD	25%	47%
Myocardial infarction	20%	0%
Peripheral vascular disease	18%	27%
Arrhythmia	18%	7%
Diabetes mellitus	15%	13%
Atherosclerosis	15%	7%
Carotid artery disease	13%	27%
Hemodialysis	11%	7%

a Mean ± sd. Medical history variables with frequency >10% in fast‐track patients reported. Fast Track: successfully completed all components of fast‐track EVAR protocol; Non‐Fast Track: failed any component of fast‐track EVAR protocol.

**Table 3:** Aortoiliac Morphology

Variable	Fast track (*n* = 114)	Non‐fast track (*n* = 15)
Proximal neck diameter (mm)^a^	23 ± 4	25 ± 10
Proximal neck angle (degrees)^a^	21 ± 20	29 ± 18
Proximal neck length (mm)^a^	25 ± 15	27 ± 13
Proximal neck calcification		
Moderate/severe	11%	8%
None/mild	89%	92%
Proximal neck thrombus		
Moderate/severe	27%	15%
None/mild	73%	85%
AAA diameter (mm)^a^	51 ± 9	52 ± 7
Left external iliac diameter (mm)^a^	7.9 ± 2.2	7.2 ± 2.3
Right external iliac diameter (mm)^a^	7.8 ± 2.1	7.5 ± 2.7

a Mean ± sd. Fast Track: successfully completed all components of fast‐track EVAR protocol; Non‐Fast Track: failed any component of fast‐track EVAR protocol.

Vascular access, stent graft delivery, and stent graft deployment were successful in all patients. Procedural outcomes favored patients who completed the fast‐track protocol (Table 4). Procedure time was 86 vs. 122 min, use of general anesthesia was 0% vs. 20%, need for intensive care unit stay was 0% vs. 13%, hospital stay was 1.1 vs. 2.1 days, and postoperative groin pain severity was 1.2 ± 1.6 vs. 4.0 ± 2.7.

**Table 4:** Procedural Data

Variable	Fast track (*n* = 114)	Non‐fast track (*n* = 15)
**Anesthesia type**		
General	0%	20%
Conscious sedation/local	96%	93%
Regional	4%	7%
**Vascular access**		
Bilateral percutaneous	100%	73%^a^
Percutaneous and cutdown	0%	13%
Cutdown	0%	13%
Contrast volume (cm^3^)^a^	125 (25–650)	142 (70–231)
Fluoroscopy time (min)^a^	18 (5–55)	23 (8–90)
Procedure time (min)^a^	86 (17–171)	122 (58–217)
Blood loss (cm^3^)^a^	45 (0–200)	50 (20–1,000)
Time to hemostasis (min)^a^	0 (0–543)	9 (0–395)
Time to normal diet (hr)^a^	6 (1–29)	10 (2–44)
Time to ambulation (hr)^a^	9 (1–25)	15 (5–48)
Intensive care unit stay	0%	13%
Hospitalization length (days)^a^	1.1 (0.6–2.7)	2.1 (1.0, 2.4)

a Median (min–max). Fast Track: successfully completed all components of fast‐track EVAR protocol; Non‐Fast Track: failed any component of fast‐track EVAR protocol.

**Figure 2:**
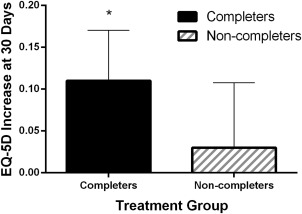
EQ‐5D change 1 month after endovascular repair. *P = 0.001 for 1‐month change; data reported as mean change and 95% confidence interval. Fast Track: successfully completed all components of fast‐track EVAR protocol; Non‐Fast Track: failed any component of fast‐track EVAR protocol.

Through the 1‐month follow‐up visit, no type I/III endoleaks, serious device‐related adverse events, AAA ruptures, surgical conversions, or AAA‐related secondary procedures were reported. One major adverse event was reported; a patient who met all study entry criteria and successfully completed the fast‐track protocol was found unresponsive on day 23 and died 5 days later due to acute respiratory failure. Overall, the 30‐day mortality rate was 0.8% and the 30‐day MAE rate was 0.8% (Table 5). Health‐related quality of life improved from 0.73 to 0.83 (*P* = 0.001) in fast‐track patients, but was largely unchanged (0.60 to 0.63, *P* = 0.70) in patients who failed any component of the fast‐track EVAR protocol (Fig. 2).

**Table 5:** Clinical Outcomes Through 1‐month Follow‐up

Variable	Fast track (*n* = 114)	Non‐fast track (*n* = 15)
Type I endoleak	0%	0%
Type III endoleak	0%	6.7%
Serious device‐related adverse event	0%	0%
Major adverse event	0.9%	0%
AAA rupture	0%	0%
Surgical conversion	0%	0%
AAA‐related secondary procedure	0%	0%
Mortality	0.9%	0%

Fast Track: successfully completed all components of fast‐track EVAR protocol; Non‐Fast Track: failed any component of fast‐track EVAR protocol.

## DISCUSSION

This interim report from the LIFE registry shows that fast‐track EVAR using a 14‐Fr endograft in well‐selected patients is feasible, safe, and results in efficient use of healthcare resources. Main findings in the 88% of patients completing all fast‐track components (PEVAR, avoidance of general anesthesia, no ICU stay, next‐day discharge) included procedure time of 86 minutes, median blood loss of 45 cm^3^, 1.1 day median hospital stay, and no type I/III endoleak, serious device‐related AEs, AAA rupture, or secondary procedures through the 1‐month follow‐up visit.

Although no control group was included in this study, the results are encouraging when compared to previous studies of traditional EVAR using the same stent graft. Mehta et al. enrolled 161 patients who underwent EVAR with the Ovation stent graft 15. In that study, 34% of patients did not require general anesthesia and 43% underwent bilateral percutaneous access. Compared to the study of Mehta, patients in the LIFE registry who completed the fast‐track protocol had comparable baseline characteristics and 1‐month clinical outcomes. Additionally, procedure time (86 vs. 110 min) and blood loss (45 cm^3^ vs. 150 cm^3^) favored those in the LIFE fast‐track arm. Considering that the Ovation stent graft has the smallest delivery profile available, it is likely that these benefits may be more pronounced compared to stent grafts with larger caliber delivery systems 10.

Importantly, adherence to strict patient selection criteria is critical to the success of a fast‐track EVAR program. Appropriate patients have no major comorbidities that would be anticipated to require intensive care support or prolong hospitalization. Additionally, femoral arteries should be free of heavy calcification or extreme tortuosity to facilitate bilateral percutaneous access. Even in less than ideal candidates, achievement of at least one of the fast‐track components may improve patient outcomes. For example, bilateral percutaneous vascular access alone results in higher technical success, less blood loss, fewer complications, and shorter hospital stay compared to surgical cutdown 10, 11, 16, 17, 18, 19, 20, 21. Avoidance of general anesthesia is associated with lower rates of mortality and morbidity and shorter intensive care unit and hospital stays compared to regional anesthesia 22, 23. Next‐day hospital discharge with no ICU stay has obvious cost benefits if patient safety is not compromised. The current clinical study is novel since these components were utilized collectively in a structured fast‐track EVAR protocol.

Limitations of this study included lack of a concurrent control group, utilization of a single endograft design, and inclusion of highly selected patients. The Ovation endograft was used exclusively in this study given the ultra low‐profile of the device makes it amenable to percutaneous access. As stent graft designs evolve toward smaller delivery profiles, adoption of fast‐track EVAR may become more widespread. Although the benefits of a fast‐track EVAR program are anticipated to be realized almost entirely within the perioperative period, the durability of outcomes beyond 1‐month follow‐up cannot be evaluated in the current study. Lastly, we did not perform a cost utility analysis for this interim report. Objective cost utility analyses comparing fast‐track EVAR to traditional EVAR are planned when final study data are available. Given that patients who complete the fast‐track EVAR protocol have excellent perioperative outcomes, it is reasonable to assume that faster procedure time, shorter hospitalization, and avoidance of ICU stay may lead to significant cost savings.

## CONCLUSION

Initial results from the LIFE study are encouraging and suggest that a fast‐track protocol is feasible, safe, and may improve efficiency of healthcare resource allocation in select patients undergoing EVAR.

## Author Contributions

- Conception and design: **ZK, VR, MH, LM**
- Data acquisition: **ZK, VR**
- Data analysis: **LM**
- Data interpretation: **ZK, VR, MH, LM**
- Drafting of manuscript: **LM**
- Critical revision of manuscript: **ZK, VR, MH**
- Final approval of the version to be published: **ZK, VR, MH, LM**
- Agreement to be accountable for all aspects of the work in ensuring that questions related to the accuracy or integrity of any part of the work are appropriately investigated and resolved: **ZK, VR, MH, LM**
